# Supplementary material for: Resolving the full spectrum of human genome variation using Linked-Reads
Source: Genome Res. 2019 Apr;29(4):635–45. doi: 10.1101/gr.234443.118 (PMC6442396; doi:10.1101/gr.234443.118)
Supplement: Supplemental Material [file supp_gr.234443.118_Supplemental_longranger-2.2.zip › longranger-2.2.tar.bz2!/lr2.2-public/lib/go/src/code.google.com/vitess/docs/index.html]

Vitess / 


Toggle navigation

- Overview
- Guides
- Reference
- Blog
- GitHub

- #### Overview

  - What is Vitess
  - Scaling MySQL with Vitess
  - Key Concepts
- #### Getting Started

  - Run Vitess on Kubernetes
    - Custom Docker Build
  - Run Vitess Locally
- #### User Guide

  - Introduction- Client Libraries- Backing Up Data- Reparenting
        - Schema Management
        - VSchema Guide
        - Schema Swap (Tutorial)
        - Sharding
          - Horizontal Sharding (Tutorial, automated)
          - Horizontal Sharding (Tutorial, manual)
          - Sharding in Kubernetes (Tutorial, automated)
          - Sharding in Kubernetes (Tutorial, manual)
        - Vitess Sequences
        - MySQL Server Protocol
        - Vitess and Replication
        - Update Stream
        - Row Based Replication
        - Topology Service
        - Transport Security Model
        - Launching
          - Scalability Philosophy
          - Production Planning
          - Server Configuration
          - 2PC Guide
          - Troubleshooting
        - Upgrading
- #### Reference Guides

  - Vitess API- vtctl Commands
- #### Other Resources

  - Presentations- Blog- Roadmap
- #### Contributing

  - Contributing to Vitess- GitHub Workflow- Code Reviews
- GitHub

Vitess is a database clustering system for horizontal scaling of MySQL.

Quickstart
Manual Build
Learn More

## Scalability

Vitess combines many important MySQL features with the scalability of a NoSQL database. Its built-in sharding features let you grow your database without adding sharding logic to your application.

## Performance

Vitess automatically rewrites queries that hurt database performance. It also uses caching mechanisms to mediate queries and prevent duplicate queries from simultaneously reaching your database.

## Manageability

Vitess automatically handles functions like master failovers and backups. It uses a lock server to track and administer servers, letting your application be blissfully ignorant of database topology.

## Connection pooling

Vitess eliminates the high-memory overhead of MySQL connections. Its gRPC-based protocol lets Vitess servers easily handle thousands of connections at once.

## Shard Management

MySQL doesn't natively support sharding, but you will likely need it as your database grows. Vitess saves you from having to add sharding logic to your app and also enables live resharding with minimal read-only downtime.

## Workflow

Vitess keeps track of all of the metadata about your cluster configuration so that the cluster view is always up-to-date and consistent for different clients.


Contact: vitess@googlegroups.com  **·**  
Announcements  **·**  
© 2017 Vitess powered by Google Inc
